# Role of Rab5 in the formation of macrophage-derived foam cell

**DOI:** 10.1186/s12944-017-0559-6

**Published:** 2017-09-12

**Authors:** Lokwern Chan, Jin Hong, Junjie Pan, Jian Li, Zhichao Wen, Haiming Shi, Jianping Ding, Xinping Luo

**Affiliations:** 1 Department of Cardiology, Huashan Hospital, Fudan University, 12 Wulumuqi Zhong Rd, Shanghai, 200040 People’s Republic of China; 2 State Key Laboratory of Molecular Biology, Institute of Biochemistry and Cell Biology, Shanghai Institutes for Biological Sciences, Chinese Academy of Sciences, 320 Yue-Yang Road, Shanghai, 200031 People’s Republic of China

**Keywords:** Rab, Foam cell, Cholesterol

## Abstract

**Background:**

Foam cells play a key role in the occurrence and pathogenesis of atherosclerosis. Its formation starts with the ingestion of oxidized low-density lipoprotein (oxLDL). The process is associated with Ras related protein in brain 5 (Rab5) which plays a critical role in regulating endocytosis and early endosomal trafficking. Base on this, we presumed that Rab5 might participate in the maturation of foam cell. The aim of this study is to investigate the effect of Rab5 on macrophage cholesterol during the evolvement of macrophage when induced by oxLDL to the formation of foam cell.

**Methods:**

Immunohistochemistry was performed to analyze the distribution of macrophages and Rab5 in atherosclerotic plaque. RNA inteference study and transfection of inactive mutant (GFP-Rab5-S34N) and active mutant (GFP-Rab5-Q79L) in U937-derived macrophage were utilized to investigate the impact of Rab5 on the process of macrophage cholesterol, which could be detected by oil red O staining, determination of intracellular lipid content, filipin staining, nile red staining and the costaining of early endosome antigen-1 (EEA-1) and 1,1′-dioctadecyl-3,3,3′,3′-tetramethylin dicarbocyanine (Dil)-labelled oxLDL (Dil-oxLDL).

**Results:**

Rab5 was found abundantly localized in macrophage rich areas of human atherosclerotic lesions. On the foam cell study, the expression of Rab5 was increased after the incubation of oxLDL. The inteference study indicated the depletion of Rab5 led to the decreases of oil red O staining areas, total cholesterol and cholesterol esters in U937-derived marophages. Moreover, the fluorescence intensity of filipin and nile red staining were lower in GFP-Rab5-S34N as compared with GFP-Rab5-Q79L. The confocal study demonstrated less Dil-oxLDL was internalized in GFP-Rab5-S34N as compared with GFP-Rab5-Q79L; the result showed also the decrease in colocalization of internalized Dil-oxLDL and EEA-1 for GFP-Rab5-S34N as compared with GFP-Rab5-Q79L.

**Conclusions:**

Rab5 plays an important role in modulating the intracellular cholesterol of macrophages and consequently mediating the formation of foam cells.

## Background

Atherosclerosis is a chronic inflammatory disease in which an artery-wall thickens as a result of the accumulation of low-density lipoprotein (LDL) cholesterol [1]. LDL particles invade endothelium and are oxidized as oxidized LDL (oxLDL) which deposits and initiates the formation of atherosclerotic plaque; Internalization of oxLDL by macrophages and smooth muscle cells leads to formation of foam cells [2].

The macrophage-derived foam cells play a key role in the occurrence and pathogenesis of atherosclerosis [3]. The formation of foam cells starts with the ingestion of oxLDL by macrophage via its scavenger receptors (SRs) [4]. Gradually, the increase in the influx and esterification of cholesterol combined with the decrease of the efflux results in the increase in the accumulation of cellular cholesterol ester (CE). The CE gathered in abundance is stored as cytoplasmic lipid droplets and leads to the formation of macrophage-derived foam cells [5]. However, the mechanism by which oxLDL enters a macrophage is still unclear.

Ras-related protein in brain 5 (Rab5) belongs to the family of Rab GTPases, which play a critical role in regulating vesicular transport and membrane-cytoskeleton interaction [6]. In addition, Rabs are essential in the transportation and fusion of endosomes which occur in the pathways of endocytosis and exocytosis. These processes are achieved by modulation of Rabs which switch between GTP-bound active form and the GDP-bound inactive form [7]. Rab5 plays a key role in receptor-mediated endocytosis [8] and particularly in early endosomal fusion [9]. Rab5 also regulates the dynamics of early endosomes [10]. Previous studies have shown that Rab5 implicates in the docking reaction of early endosomes to lipid droplets [11]. Whether Rab5 is involved in facilitating the entry of oxLDL into macrophages and the formation of foam cell is not yet reported so far.

With its function in the endocytosis and early endosomal trafficking, we presumed that Rab5 might participate in the regulation of the delivery of cholesterol and thus affects the formation of foam cell.

In this study, we investigated the effect of Rab5 on macrophage cholesterol during the evolvement of macrophage when induced by oxLDL to the formation of foam cell. Based on the investigation, we could establish a connection between Rab5 and the formation of foam cells.

## Methods

### Immunohistochemistry

Formalin-fixed, paraffin-embedded sections were subjected to immunohistochemical staining using primary antibodies against Rab5 (rabbit anti-human IgG monoclonal antibodies, Cell Signaling Technology, USA) and CD68 (Goodbio Technology, Wuhan, China). Briefly, the sections were deparaffinized and rehydrated in a graded series of ethanol concentration. For antigen retrieval, the sections were heated in an EDTA buffer (pH 9.0). After that, the sections were incubated with 3% H_2_O_2_ in the dark for 25 min to quench the activity of endogenous peroxidase. After blocking with 3% bovine serum albumin (BSA), the primary antibody was added in the indicated dilutions, and then incubated overnight at 4 °C. After washing, the sections were treated with biotinlylated second antibody at room temperature for 50 min. Then, horseradish peroxidase (HRP) was applied and the antigen signal area was developed by 3,3-diaminobenzidine (DAB). All sections were counterstained with haematoxylin. Images were captured using Pannoramic 250 FLASH (3D Histech, Hungary) and a C2^+^ confocal microscopy (Nikon Instruments, Japan).

### Cell culture

Human monocytic leukemic cell line U937 was obtained from the cell bank of Shanghai Institute of Biological Sciences, the Chinese Academy of Sciences. U937 cells were cultured in RPMI 1640 (Hyclone, USA) with 10% (*v*/v) fetal bovine serum (FBS) (Zhejiang Tianhang Biotechnology, China) at 37 °C in a 5% CO_2_ humidified incubator. For each experiment, equal amount (3 × 10^5^cells/ml) of U937 cells were incubated with 100 nmol/ml Phorbol 12-myristate 13-acetate (PMA) (Sigma Adrich, USA) for 72 h to differentiate into macrophages. Cells were then cultured for another 48 h without PMA. More than 90% of the cells adhered to dishes, which demonstrated that most of the cells had differentiated into macrophages. Non-adherent cells were washed off with PBS and only adherent cells were used for further study.

### LDL isolation and modification

oxLDL was purchased from Yiyuan biotechnologies (Guangzhou, China). Briefly, the LDL was isolated from blood-bank-produced human plasma by ultra-centrifugation (1.019–1.063 g/ml). For the preparation of oxLDL, LDL was oxidized using 5 μM Cu_2_SO_4_ (oxidant) in PBS at 37 °C for 20 h. Following that, oxidation was terminated by adding excess EDTA-Na_2_. The extent of modification was analyzed on agarose gel electrophoresis [12, 13]. The oxLDL used in this study migrated 2 fold further than the native LDL.

### Oil red O staining

Cells were washed 2 times with PBS and fixed with 4% paraformaldehyde at room temperature for 20 min. After washing 2 times with deionized water, the cells were stained with 0.3% Oil red O solution (dissolved in isopropanol:water, 3:2) at 37 °C for 3 h. The Oil red O solution was removed and the cells were washed with deionized water for 3 times. Prior to sealing with neutral gum, the slides were stained with haematoxylin. Images were captured using an Olympus IX73 inverted microscope.

### Determination of intracellular lipid content

The extraction process was based on the method described by Bligh and Dyer [14]. Briefly, after extensive wash, the cells were pelleted and resuspended in a lysis buffer (Beyotime, China) containing 1 mM phenylmethanesulfonylfluoride (PMSF). The supernatant was collected after centrifugation at 12000 rpm for 15 min at 4 °C. Protein concentrations were measured using the Enchanced BCA Protein Assay Kit (Beyotime, China). 100 μl of lysate was placed in a microtube, and then 125 μl of chloroform and 250 μl of methanol were added. The mixture was vortexed for 1 min. The apolar and polar phases were divided by the addition of 125 μl of chloroform and 125 μl of distilled water. The mixture was centrifuged at 1000 rpm at room temperature for 5 min. The lower lipid containing phase was recovered and transferred to a clean microtube. Total cholesterol (TC) and free cholesterol (FC) contents were determined using the Cholesterol Quantification Kit (Sigma Aldrich, USA) and measured using a BioTek Synergy NEO (BioTek Instrument, USA) at the absorbance of 570 nm. CE was determined as the difference between TC and FC (TC minus FC) and was expressed in units of mg/g cellular protein.

### Quantitative RT/PCR (qRT-PCR)

Total cellular RNA was extracted using the RNAprep Pure Cell/Backeria Kit (Tiangen Biotech, Beijing, China) according to the manufacturer’s instructions, and the complementary DNA (cDNA) was synthesized using a PrimeScript™ RT Master Mix (Takara, Japan). qRT-PCR was carried out using a SYBR Premix Ex Taq™ II (Takara, Japan) and performed according to the manufacturer’s instructions. The reactions were performed for 40 cycles of 95 °C for denaturation and 60 °C for annealing and extension using an ABI 7500 real-time PCR system (Applied BioSystem, USA). The relative amount of mRNA was calculated with the β-actin (ACTB) mRNA as the invariant control. The primer sequences used were: Rab5, 5′-TGTGGACACTTGTTTCATTGG-3′ and 5′-GTGGAGAAATGGGCTGGTTA-3′. ACTB, 5′-CCTGGCACCCAGCACAAT-3′ and 5′-GGGCCGGACTCGTCATAC-3′. Fold induction values were calculated using the 2^∆∆Ct^ method. The abundance of mRNA was normalized with ACTB, and the value was expressed as “relative gene expression”.

### Western blot

Cells were lysed with a lysis buffer containing 1 mM PMSF. The supernatant was obtained after centrifugation at 12000 rpm for 15 min at 4 °C. After measurement of the protein concentration, the samples were mixed with a loading buffer and boiled for 8 min. The samples were then electrophoresed through a 5–12% Tris-HCl SDS-PAGE (110 V, 1.5 h) and transferred to Immobilon-P PVDF Transfer Membranes (Millipore, USA) with 100 V for 50 min. The membranes were blocked in a TBS-T buffer containing 5% nonfat milk at room temperature for 1 h, and then incubated accordingly with rabbit anti-human Rab5 antibody (Cell Signaling Technology, USA) at 4 °C overnight. After washing with the TBS-T buffer, the filter was incubated with goat anti-rabbit horseradish peroxidase antibody (Jackson ImmunoResearch Laboratories, USA) at a concentration of 1:5000 for 1 h. Immobilon Western Chemiluminescent HRP Substrate (Millipore, USA) was used to detect these proteins and exposed to ImageQuant LAS 4000 (GE Healthcare Life Sciences, USA).

### RNA interference

Duplexes of siRNAs were synthesized by GenePharma (Shanghai, China). Three different oligonucleotides were composed by GenePharma and were used to silence Rab5. The three different sequences of the siRNAs were as follows: the first sequence comprised of siRab5–661 siRNA sense strand 5′-GCC AAU UUC AUG AAU UUC ATT-3′, with antisense of 5′-UGA AAU UCA UGA AAU UGG CTT-3′; the second sequence comprised of siRab5–775 sense 5′-CAG CCA UAG UUG UAU AUG ATT-3′, with antisense of 5′-UCA UAU ACA ACU AUG GCU GTT-3′; the third sequence comprised of siRab5–938 sense 5′-GUC CUA UGC AGA UGA CAA UTT-3′, with antisense is 5′-AUU GUC AUC UGC AUA GGA CTT-3′. The negative control (siCTL) siRNA sense strand is 5′- UUC UCC GAA CGU GUC ACG UTT-3′, with antisense of 5′-ACG UGA CAC GUU CGG AGA ATT-3′. For transfection, cells were transfected with Lipofectamine 2000 (Life Technologies, USA) according to the manufacturer’s instructions. The transfection effect was observed under a fluorescent microscopy (Olympus IX73 inverted microscope). The gene silencing effect was analyzed by qRT-PCR 48 h after transfection.

### Cell Transfection

Rab5 mutants were described elsewhere [15]. The pEGFP-Rab5-S34N and pEGFP-Rab5-Q79L were constructed by cloning Rab5-S34N and Rab5-Q79L into the EcoRI/BamHI sites of GV144 (Genechem, Shanghai, China), and verified by sequencing. Transfection was performed using Lipofectamine 2000 (Life Technologies, USA) according to the manufacturer’s instructions. For each transfection, 0.5 μg/well of DNA was used in 24-well plates. Experiments were carried out 72 h after transfection.

### Immunofluorescence and confocal microscopy

Cells were washed with PBS and fixed with 4% paraformaldehyde for 15 min at room temperature. After washing 3 times with PBS, intracellular FC and CE was visualized using filipin (Sigma Adrich, USA) and nile red (Life Technologies, USA) staining, respectively. For filipin staining, a fresh 5 mg/ml filipin stock solution prepared in DMSO was diluted to a concentration of 50 μg/ml with PBS containing 10% FBS. The cells were stained with 50 μg/ml filipin solution in the dark for 30 min. For nile red staining, 10 mg/ml nile red stock solution prepared in acetone was diluted at 1:10,000 in PBS as working solution. The cells were stained with 1 μg/ml nile red solution in the dark for 30 min. The solution was removed and the cells were mounted and then washed 3 times with PBS. Filipin and nile red signals of stained cells were analyzed using an Olympus IX73 inverted microscope at excitation wavelength of 320 nm and 552 nm, respectively. The distribution and level of intracellular oxLDL were analyzed after 3 h incubation of 40 μg/ml 1,1′-dioctadecyl-3,3,3′,3′-tetramethylin dicarbocyanine (Dil)-labelled oxLDL (Dil-oxLDL) followed by fixation with 4% paraformaldehyde. Images were acquired using a Pannoramic MIDI using at excitation wavelength of 549 nm. The fluorescence in GFP-expressing cells was quantified by image processing using an Image-Pro Plus (Media Cybernetic, USA).

For colocalization study, Dil-oxLDL and immunostaining of early endosomal antigen-1 (EEA-1) were determined to analyze the colocalization of oxLDL and early endosome. Following Dil-oxLDL treatment, the cells were washed 3 times with PBS and fixed with 4% paraformaldehyde in PBS at room temperature for 10 min, and then washed 3 times again with PBS. The fixed cells were permeabilized with 0.1% Triton X-100. After a further wash, the cells were blocked with 2% BSA for 1 h. Mouse anti-human EEA-1 primary antibody (BD Biosciences, USA) was diluted in 2% BSA at a concentration of 1:100 and applied for 3 h at room temperature. The cells were washed 3 times before labeling with secondary antibody, Dylight 405 (Jackson ImmunoResearch Laboratories, USA). After 3 times washing with PBS, the cells were mounted and viewed using a C2^+^ confocal microscopy (Nikon Instruments, Japan). An Image-Pro Plus (Media Cybernetic, USA) was used for the quantification of colocalization. The Pearson’s correlation coefficient (PCC) gives a measure of the overlap between two fluorescence signals. It was calculated by analyzing 20–30 cells and expressed as the mean with standard deviation (SD).

### Statistical analysis

Results are reported as means ± SD. The group *t*-test and Wilcoxon’s test were applied for the assessment of the results of qRT-PCR and the fluorescence intensity of the immunofluorescence study between the groups were analyzed. The PCC was used for the quantification of colocalization study of internalized Dil-oxLDL and EEA-1 in U937-derived macrophages with *p* < 0.05 was considered statistically significant. The statistical software package Stata 11.0 (Stata Corp. LP, USA) was used for the analysis.

## Results

### Rab5 was found abundantly localized in macrophage rich areas of human atherosclerotic lesions

**Fig. 1:**
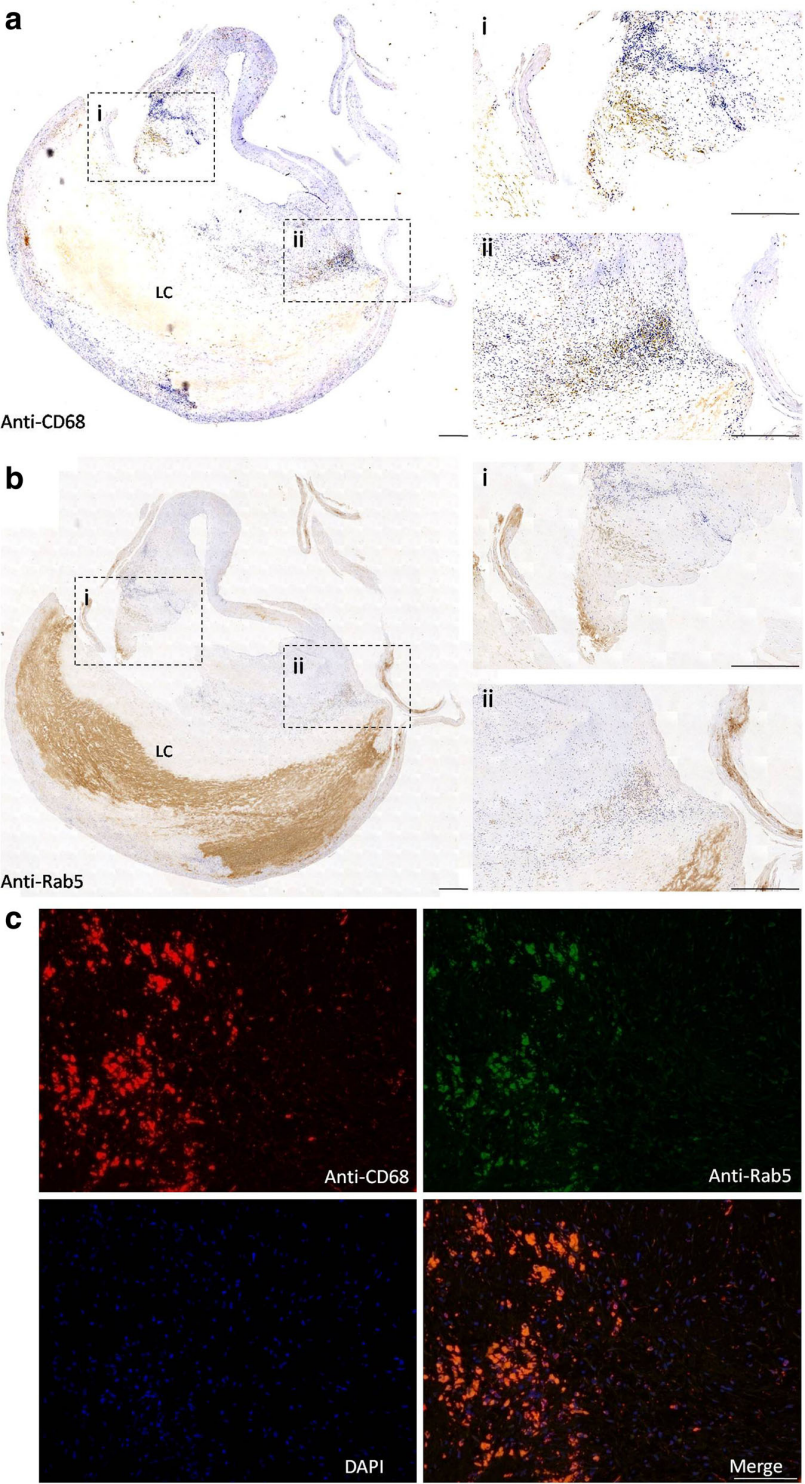
Rab5 was localized in abundance in the macrophage rich regions of human atherosclerotic lesions. **a** Immunohistochemical staining of sections of atheromatous human carotid artery with anti-CD68 antibody and **b** anti-Rab5 antibody. Scale bars = 500 μm. **c** Colocalization of anti-CD68 and anti-Rab5 antibodies. Scale bars = 100 μm

To determine the distribution of macrophages and Rab5 in human atherosclerotic plaque, we stained sections of atheromatous human carotid arteries with anti-CD68 and anti-Rab5 antibodies. We found that anti-CD68 antibody was markedly distributed in cells surrounding lipid core (LC) (Fig. 1a), indicating the infiltration of macrophage to the atheroma plaque. Furthermore, Rab5 staining exhibited that the majority of Rab5 was localized in macrophage rich areas (Fig. 1b), overlapping with the CD68-positive areas as mentioned above. Moreover, the co-staining results also showed that the anti-68 antibody staining areas overlapped significantly with the anti-Rab5 staining areas (Fig. 1c), demonstrating that the Rab5 positive cells were actually macrophages, and Rab5 was intensively expressed in macrophages. In addition, we also observed that Rab5 was heavily exhibited in the arterial intima.

### Expression of Rab5 in U937-macropages and foam cells

**Fig. 2:**
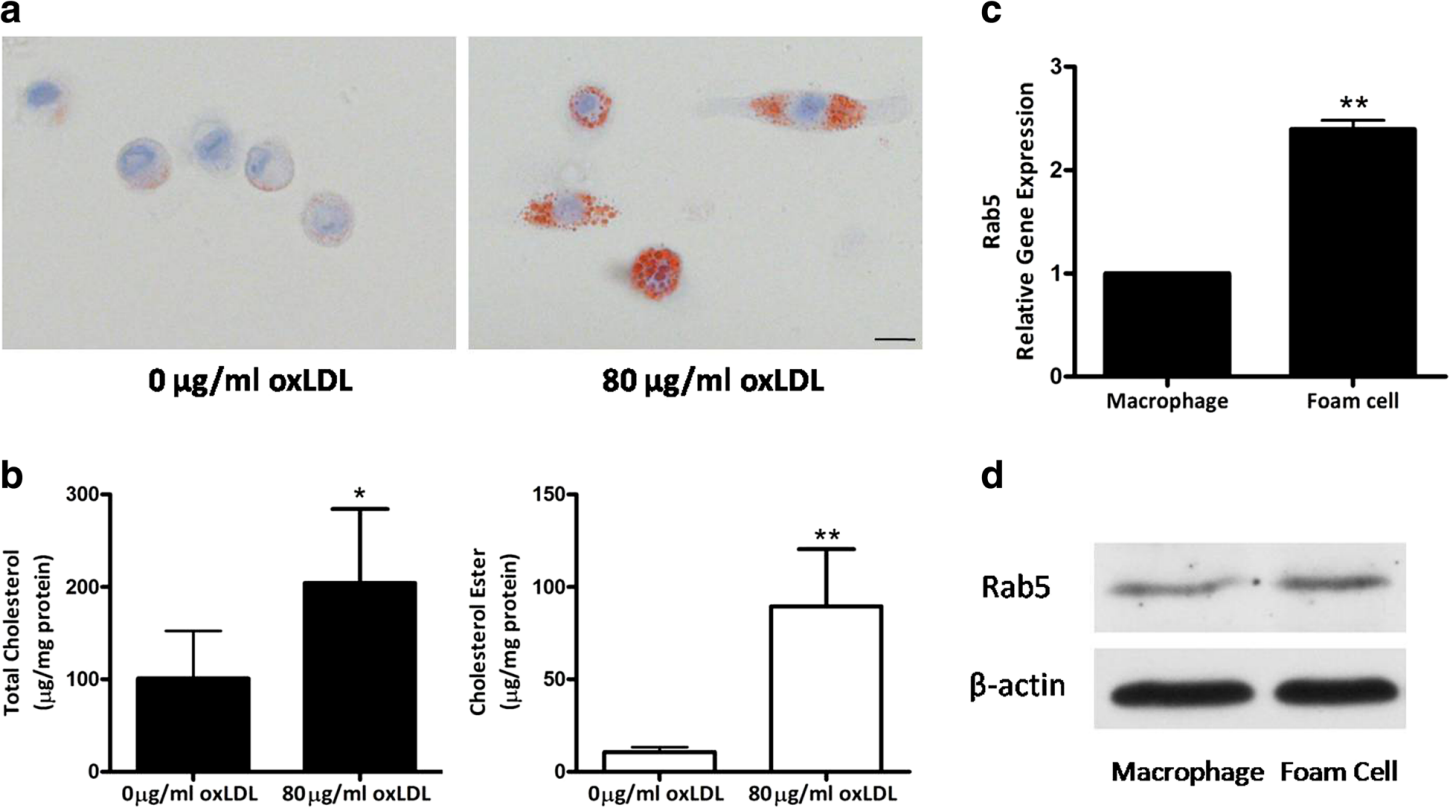
Effect of Rab5 on foam cell formation in U937-derived macrophages. **a** Oil red staining for U937-derived macrophages following 48 h incubation with 80 μg/ml oxLDL. Scale bars = 20 μm. **b** Quantification of TC and CE of the U937-derived macrophages after treated with 80 μg/ml oxLDL (*n* = 4, *P < 0.05; **P < 0.01 vs. 0 μg/ml oxLDL treated control). **c** Real time PCR of Rab5 mRNA expression (*n* = 3, *P < 0.05). **d** Western Blot analysis of Rab5 expression followed by oxLDL treatment

Most of the U937 cells were adhered to dishes in the presence of PMA, indicating that most cells had differentiated into U937-derived macrophages. The cells were then incubated with oxLDL to induce the formation of foam cell. Following with oxLDL treatment, the U937-derived macrophages underwent obvious morphologic changes with significant increase in lipid droplet accumulation in macrophages as analyzed using oil red O staining (Fig. 2a). In addition, the quantification results indicated a significant increase in TC and CE in the U937-derived macrophages after the treatment of oxLDL (Fig. 2b). These results revealed that the U937-derived macrophages were transformed into the foam cells.

In order to evaluate the effect of Rab5 on the foam cell formation, the expression of Rab5 was detected during the maturation of macrophage-derived foam cells. RT-PCR showed that the expression of Rab5 mRNA was significantly increased at the 48th hour after the incubation of oxLDL, the increase of foam cells was 2.4 times more than macrophages (Fig. 2c). Consistently, Rab5 protein levels were increased following with oxLDL treatment (Fig. 2d).

### siRab5–775 caused higher Rab5 gene down-regulation

**Fig. 3:**
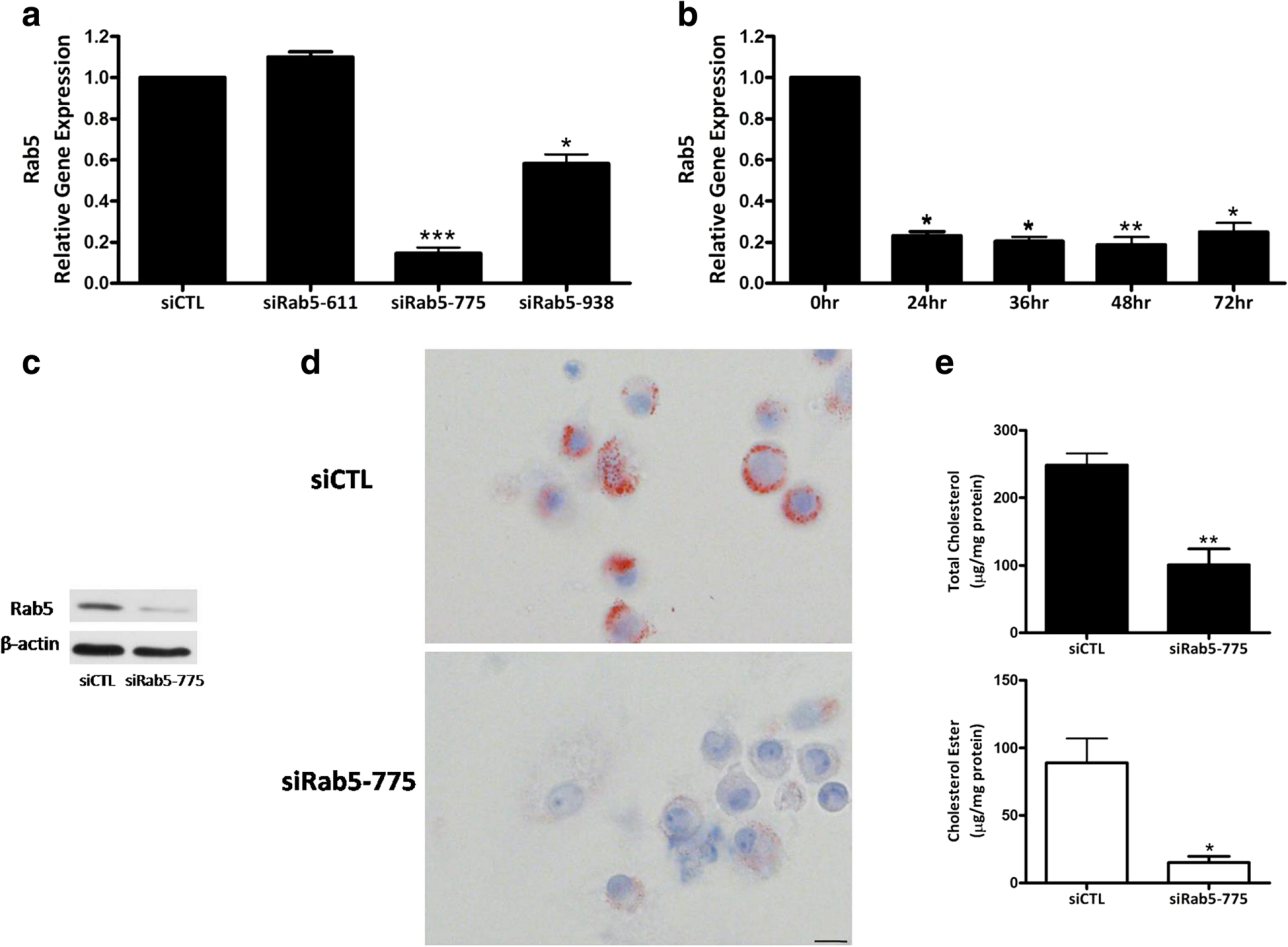
Down-regulation of Rab5 in U937-derived macrophages inhibited the formation of foam cells. **a** U937-derived macrophages were lysed and analyzed by qRT-PCR for Rab5 mRNA after transfection with the indicated siRNA (*n* = 2, *P < 0.05; ***P < 0.001). **b** Time course study was carried out to evaluate the expression of Rab5 mRNA followed by the transfection of siRab5–775 (n = 3, *P < 0.05; ***P < 0.001 vs. 0 h control group). **c** Efficiency of knock-down of Rab5 protein by siRab5–775 was analyzed by western blot. **d** U937-derived macrophages transfected with the indicated siRNAs for 48 h, incubated for 48 h with 80 μg/ml oxLDL, and stained with oil red O. Scale bars = 20 μm. **e** Quantification of TC and CE of U937-derived macrophages treated with indicated siRNAs and 80 μg/ml oxLDL (n = 3, *P < 0.05; **P < 0.01)

qRT-PCR analysis indicated that siRab5–775 caused significant down-regulation of Rab5, which led to a 90% decrease in the level of Rab5 mRNA (Fig. 3a). Furthermore, time course analysis was performed to investigate the efficacy of siRab5–775 in silencing the expression of Rab5 in the U937-derived macrophages. The results showed that the down-regulating effect of Rab5 was occurred at 24 h after the administration of siRab5–775, which reached the maximum at the 48th hour and continued for at least 72 h following the transfection of siRab5–775 (Fig. 3b). The expression levels of Rab5 protein were inhibited followed by the transfection of siRab5–775, which were confirmed by the analysis of western blot (Fig. 3c).

### Depletion of Rab5 in U937-derived macrophages inhibited foam cell formation

To further confirm the effect of Rab5 on the lipid droplet and the foam cell formation from U937-derived macrophages, the impact of Rab5 depletion on foam cell maturation in U937-derived macrophages was investigated following oxLDL induction. We found that the U937-derived macrophages treated with siRab5–775 showed lower percentage of oil red O positive areas compared to the U937-derived macrophages treated with siCTL (Fig. 3d). Correspondingly, the TC and CE was significantly decreased in siRab5–775 treated U937-derived macrophages compared with that of siCTL treated macrophages (Fig. 3e).

### Inactive GDP-bound Rab5 reduced the level of FC and CE in U937-derived macrophages

**Fig. 4:**
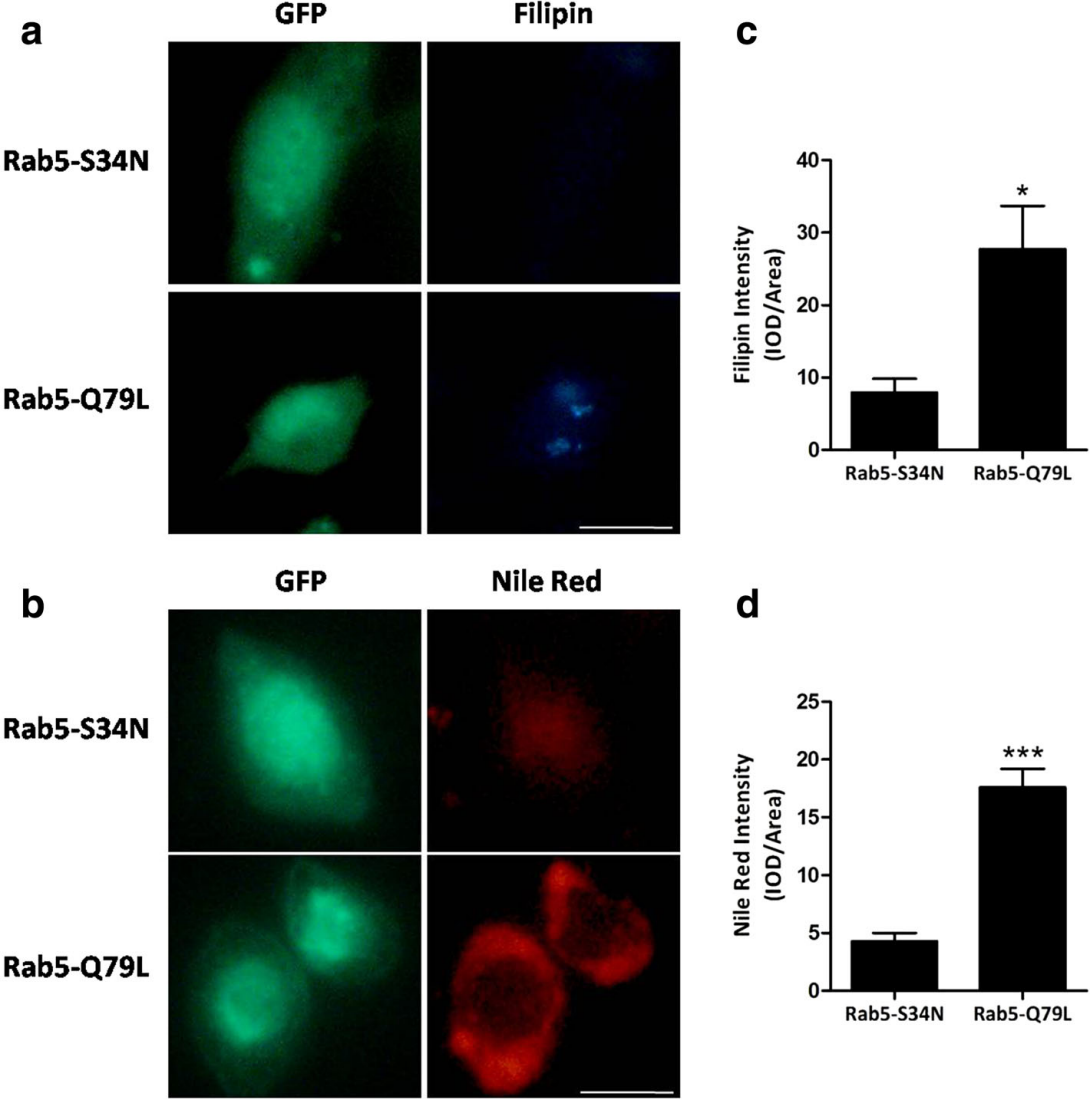
Inactive GDP-bound Rab5 reduced the level of FC and CE. **a**, **b**, **c** and **d** U937-derived macrophages were transfected for 72 h with plasmids encoding GFP-tagged inactive or active mutated forms of Rab5 as indicated. **a** Cells were incubated for 3 h with 80 μg/ml oxLDL and stained with filipin. Scale bars = 10 μm. **b** Cells were treated for 24 h with 80 μg/ml oxLDL and stained with nile red. Scale bars = 10 μm. **b** and **d** Fluorescence intensity of filipin and nile red was quantified following oxLDL incubation (*n* = 50, *P < 0.05; ***P < 0.001)

We hereby introduced the constitutively inactive and active mutants of GFP-tagged Rab5 to characterize the effect of Rab5 in regulating the distribution of intracellular FC and CE. In this study, the intracellular FC was detected by filipin staining after 3 h incubation of oxLDL, whereas CE was detected by nile red staining following the incubation of oxLDL for 24 h. We found that intracellular FC and CE was lower in the U937-derived macrophages expressing the inactive GDP-bound Rab5 mutant (GFP-Rab5-S34N) as compared with the U937-derived macrophages expressing the active GTP-bound Rab5 mutant (GFP-Rab5-Q79L) (Fig. 4a and b). Furthermore, the quantitative analysis of FC and CE showed lower degree of fluorescence intensity in GFP-Rab5-S34N than in GFP-Rab5-Q79L (Fig. 4c, d).

### Inactive GDP-bound Rab5 impaired oxLDL internalization and disrupted the interaction between oxLDL and early endosomes in U937-derived macrophages

**Fig. 5:**
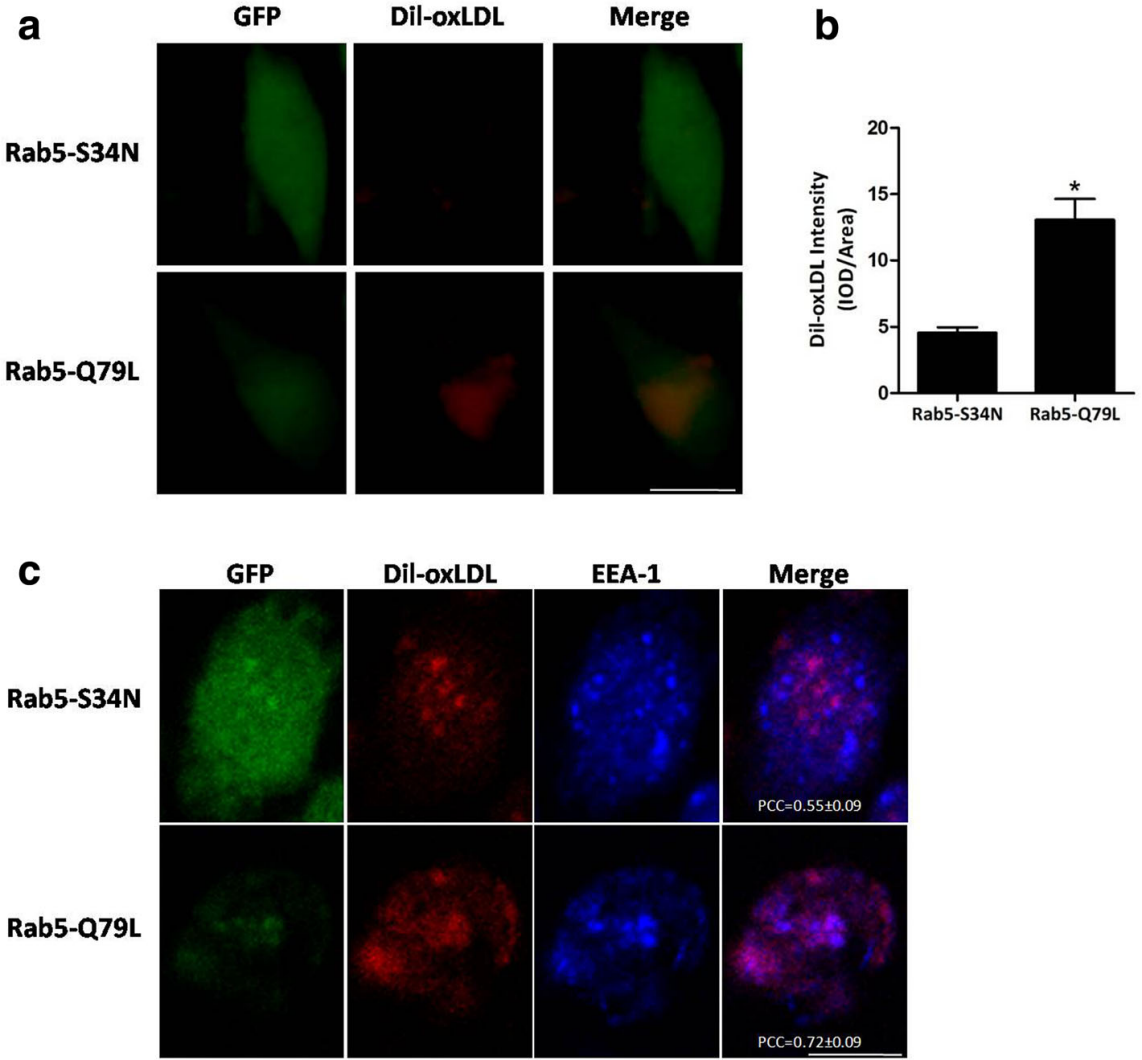
Inactive GDP-bound Rab5 impaired oxLDL internalization and disrupted the interaction between oxLDL and early endosomes in U937-derived macrophages. **a**, **b** and **c** U937-derived macrophages were transfected for 72 h with plasmids encoding GFP-Rab5-S34N and GFP-Rab5-Q79L as indicated. **a** Cells were loaded for 3 h with 40 μg/ml Dil-oxLDL before fixation. Scale bars = 10 μm. **b** Quantification of intracellular Dil-oxLDL was assayed after the incubation of Dil-oxLDL (n = 50, *P < 0.05). **c** Colocalization of internalized Dil-oxLDL and EEA-1 in U937-derived macrophages transfected with GFP-Rab5-S34N and GFP-Rab5-Q79L. Scale bars = 10 μm. The means ± SD of the PCC of 20–30 cells are shown

We utilized fluorescence-labeled oxLDL (Dil-oxLDL) to examine the impact of Rab5 on the internalization of oxLDL in U937-derived macrophages. We found that lesser Dil-oxLDL was internalized in GFP-Rab5-S34N as compared with GFP-Rab5-Q79L, after both had undergone a continuous uptake of Dil-oxLDL for 3 h (Fig. 5a). The quantitative results showed that the U937-derived macrophages with GFP-Rab5-Q79L exhibited higher intensity of Dil-oxLDL than the U937-derived macrophages with GFP-Rab5-S34N (Fig. 5b). However, our experiments also indicated that the internalization of oxLDL was not totally blocked in the U937-derived macrophages with GFP-Rab5-S34N. Similar results were observed in our colocalization study of Dil-oxLDL and EEA-1 in which the impact of Rab5 in regulating the interaction of oxLDL and early endosomes in U937-derived macrophages was analyzed. We observed that the colocalization of internalized Dil-oxLDL and EEA-1 decreased in GFP-Rab5-S34N as compared with GFP-Rab5-Q79L (Fig. 5c). In addition, the quantified results showed that the PCC was lower in the U937-derived macrophages with GFP-Rab5-S34N than those with GFP-Rab5-Q79L.

## Discussion

Rab5 is an important regulator of the early endocytic pathway [16], which modulates endocytosis and mediates membrane traffic into early endosomes [17]. In addition, Rab5 is a key modulator of early endosomal formation and maturation that regulates the conversion of early endosomes into late endosomes and lysosomes [18]. Previous studies found that the disruption of Rab5 impedes the synthesis of PI(3)P. Based on the similar ground, and because of the importance of Rab5 in regulating PI3K/mTOR pathway, the disruption of Rab5 also resulted in the suppression on mTORC [19]. The effect was supported by Nielson et al., whose study found that Rab5 regulates movements of early endosomes on microtubules and the regulating movements depended on the activation of PI(3)K through Rab5 [20].

Foam cell formation is related to the disturbance of intracellular cholesterol processing, and the disturbance comprised the disruption of the uptake of oxLDL, dysregulation of the internalized cholesterol and dysfunction of cholesterol efflux, all of which ultimately resulted in the accumulation of intracellular CE [3]. On the consideration that Rab5 plays a critical role in regulating the fusion between endocytic vesicles and early endosomes, we proceed further to investigate the role of Rab5 in the formation of macrophage-derived foam cells.

Based on the research on immnunohistochemistry which was performed to analyze the distribution of macrophages and Rab5 in atherosclerotic plaque, we found that Rab5 was highly expressed in macrophage rich areas, surrounding the lipid core of atheroma plaque, suggesting that the accumulation of Rab5 in macrophages may lead to the progression of atheroslerosis. Moreover, since the majority of Rab5 exhibited in the arterial intima, it is possible that Rab5 initiated the dysfunction of endothelium.

Our study on foam cell maturation revealed that U937-derived macrophages produced significant increase in the accumulation of lipid droplet following the incubation of oxLDL as demonstrated by oil red O staining. Correspondingly, the quantitative results showed marked increase of TC and CE followed by the treatment of oxLDL. Taken together, our results demonstrated that lipids were uptaken by U937-derived macrophages and the macrophages were then differentiated into foam cells. In addition, we found that during the formation of macrophage-derived foam cells, the expression levels of Rab5 mRNA and protein were increased. These findings showed that Rab5 played an important role in the maturation of foam cells. Previous study demonstrated the critical role of PI3K signaling played in the uptake of modified LDL via its up-regulation effect on SRs, which in turn affects the maturation of foam cells [21]. Based on the analysis mentioned as above, the formation of foam cells may be driven by the association of Rab5 with PI3K pathway.

Among the three siRNA sequences comprising of siRab5–775, siRab5–611 and siRab5–938, siRab5–775 achieved the highest gene-silencing effect on Rab5 expression. The down-regulation effect of Rab5 brought by siRab5–775 began at the 24th hour, peaked at the 48th hour, and continued its effect for more than 72 h after transfection. According to Zeigerer et al., the down-regulation of Rab5 inhibits the uptake of LDL in primary hepatocytes, resulting in marked reduction of early endosomes, late endosomes and lysosomes in hepatocytes [22]. Our study showed that after the incubation of oxLDL, the levels of TC and CE were lower in macrophages treated by siRab5–775 as compared with macrophages treated by siCTL. Consequently, the down-regulation of Rab5 in macrophages led to the suppression of CE accumulation and attenuated the formation of oxLDL-induce foam cells. This effect may be triggered by the impairment of the interaction between early endosomes and lipid droplets because according to previous study the binding of early endosomes to lipid droplets depends on the mediation of Rab5 [11].

To further confirm the role of Rab5 in macrophage cholesterol processing, we utilized two types of mutants, comprising the constitutively inactive mutant, GFP-Rab5-S34N and active mutant, GFP-Rab5-Q79L. The experimental results showed that following with oxLDL treatment, the levels of intracellular FC and CE were lower in GFP-Rab5-S34N as compared with GFP-Rab5-Q79L. The reason is because less amount of internalized Dil-oxLDL was detected in GFP-Rab5-S34N, corresponding to less amount of Dil-oxLDL being uptaken by Rab5-GDP during its endocytosis. Secondly, the colocalization of internalized Dil-oxLDL and early endosome marker EEA-1 was disrupted in the U937-derived marcrophages with GFP-Rab5-S34N, demonstrating that the interaction of oxLDL with early endosome was impaired, and thus disrupted the trafficking of oxLDL in early endosomes.

In this study we found that the regulation of Rab5 in facilitating the internalization of oxLDL and the transportation of oxLDL in early endosomes consequently promoted the formation of macrophage-derived foam cell. On the other hand, Dwived et al. found that autophagy is activated when the regulatory gene of the endocytic pathway, Rab5 is disrupted. The authors hypothesized that autophagy took over the function of the endocytic pathway whenever malfunctioning of the endocytic pathway occurred. The hypothesis suggested that Rab5 acted like a switch to regulate the process between autophagy and endocytic pathway [23]. Furthermore, according to previous researches, autophagy was also involved in the process of lipophagy, where autophagy plays a critical part in regulating intracellular lipid contents [24–26]. Based on some reasoning mentioned above, we conjectured that whenever Rab5 ceases to function, the process of lipophagy is activated to take over the function of the disrupted endocytic pathway of Rab5. Such action leads to the further decrease of TC and CE in Rab5 silenced U937-derived macrophages. Nonetheless, further studies are necessary in order to fully understand the exact function of Rab5 in regulating the cholesterol of macrophages.

## Conclusions

In conclusion, our study demonstrated that Rab5 plays an important role in modulating the intracellular cholesterol of macrophages and thus mediating the formation of foam cells. This process is accomplished through the regulation of the endocytosis and early endosomal trafficking by Rab5.

## Abbreviations

BSA: Bovine serum albumin
cDNA: Complementary DNA
CE: Cholesterol ester
DAB: 3,3-diaminobenzidine
Dil-oxLDL: 1,1′-dioctadecyl-3,3,3′,3′-tetramethylin dicarbocyanine (Dil)-labelled oxLDL
EEA-1: Early endosome antigen-1
FBS: Fetal bovine serum
FC: Free cholesterol
HRP: Horseradish peroxidase
LC: Lipid core
LDL: Low-density lipoprotein
oxLDL: Oxidized low-density lipoprotein
PMA: Phorbol 12-myristate 13-acetate
PMSF: Phenylmethanesulfonylfluoride
Rab5: Ras related protein in brain 5
siCTL: negative control
SRs: Scavenger receptors
